# The association between dynamic lung volume and peak oxygen uptake in a healthy general population: the HUNT study

**DOI:** 10.1186/s12890-018-0762-x

**Published:** 2019-01-06

**Authors:** Øystein Rasch-Halvorsen, Erlend Hassel, Arnulf Langhammer, Ben M. Brumpton, Sigurd Steinshamn

**Affiliations:** 1 K.G. Jebsen Center for Exercise in Medicine, Department of Circulation and Medical Imaging, Faculty of Medicine and Health Sciences, NTNU, Norwegian University of Science and Technology, Trondheim, Norway; 2 Department of Public Health and Nursing, Faculty of Medicine and Health Sciences, NTNU, Norwegian University of Science and Technology, Trondheim, Norway; 3 Clinic of Thoracic and Occupational Medicine, St. Olavs Hospital, Trondheim University Hospital, Trondheim, Norway; 4 K.G. Jebsen Center for Genetic Epidemiology, Department of Public Health and Nursing, Faculty of Medicine and Health Sciences, NTNU, Norwegian University of Science and Technology, Trondheim, Norway; 5 NTNU, Faculty of Medicine and Health Sciences, Department of Circulation and Medical Imaging, 8905, 7491 Trondheim, Norway

**Keywords:** Exercise test, Oxygen consumption, Respiratory function tests, Forced expiratory volume

## Abstract

**Background:**

Although dynamic lung volume is not considered a limiting factor of peak oxygen uptake (VO_2peak_) in healthy subjects, an association between forced expiratory lung volume in one second (FEV_1_) and VO_2peak_ has been reported in a healthy population aged 69 – 77 years. We hypothesized that a corresponding association could be found in a healthy general population including young and middle-aged subjects.

**Methods:**

In a population-based study in Norway, we investigated the association between FEV_1_ above the lower limit of normal (LLN) and VO_2peak_ using linear regression and assessed the ventilatory reserve (VR) in healthy subjects aged 20 – 79 years (*n* = 741).

**Results:**

On average, one standard deviation (SD) increase in FEV_1_ was associated with 1.2 ml/kg/min (95% CI 0.7 – 1.6) higher VO_2peak_. The association did not differ statistically by sex (*p*-value for interaction = 0.16) and was similar (0.9 ml/kg/min, 95% CI 0.2 – 1.5) in a sensitivity analysis including only never-smokers (*n* = 376). In subjects below and above 45 years of age, corresponding estimates were 1.2 ml/kg/min (95% CI 0.5 – 1.8) and 1.2 ml/kg/min (95% CI 0.5 – 1.9), respectively. Preserved VR (≥ 20%) was observed in 66.6% of men and 86.4% of women.

**Conclusions:**

Normal dynamic lung volume, defined as FEV_1_ above LLN, was positively associated with VO_2peak_ in both men and women, in never-smokers and in subjects below and above 45 years of age. The majority of subjects had preserved VR, and the results suggest that FEV_1_ within normal limits may influence VO_2peak_ in healthy subjects even when no ventilatory limitation to exercise is evident.

## Background

Peak oxygen uptake (VO_2peak_) is an indicator of cardiorespiratory fitness (CRF) and a primary measure in cardiopulmonary exercise testing (CPET). The VO_2peak_ achieved at CPET is determined by the efficiency of the integrated oxygen transport and utilization system during maximal exercise [1].

The upper functional limit of the cardiovascular system is generally accepted as the primary limiting factor of VO_2peak_ in healthy subjects [2–4]. More specifically, the maximal cardiac stroke volume is a major factor determining the maximal capacity for oxygen transport during exercise [3]. In contrast, the functional reserves of the healthy pulmonary system for ventilation and gas exchange are considered well preserved in non-athletes exercising at sea level [5, 6]. The impact of dynamic lung volume on VO_2peak_ is commonly assessed indirectly by the ventilatory reserve (VR), reflecting the difference between estimated ventilatory capacity and measured minute ventilation at peak exercise (VE_peak_). Reduced VR is considered indicative of ventilatory limitation [7].

Although normal dynamic lung volume is not the primary limiting factor of VO_2peak_, forced expiratory lung volume in one second (FEV_1_) above the lower limit of normal (LLN) was recently found to be positively associated with VO_2peak_ in healthy elderly subjects [8]. However, this has not been studied in young and middle- aged subjects, and the potential influence of FEV_1_ above LLN on VO_2peak_ is largely unknown. In healthy subjects, CRF and VO_2peak_ are associated with risk of all-cause mortality [9]. Therefore, any influence of FEV_1_ on the overall efficiency of the oxygen transport chain could be important.

We aimed to investigate the association between normal dynamic lung volume and CRF in a general population and hypothesized that FEV_1_ above LLN is associated with VO_2peak_ in healthy subjects of a wide age-span. Additionally, we evaluated the VR and explored whether an association between FEV_1_ above LLN and VO_2peak_ differs by sex. Provided the finding of an association between FEV_1_ above LLN and VO_2peak_, we also hypothesized an association between FEV_1_ above LLN and peak oxygen pulse (O_2pulse_), a proposed non-invasive estimator of cardiac stroke volume [10].

## Methods

### Study population

The Nord-Trøndelag Health Study (HUNT) is a population-based cohort study in Nord-Trøndelag County in Norway. The adult part of the third survey (HUNT3), conducted between October 2006 and June 2008, invited all residents aged 20 years or more (*n* = 93,860) and 54.1% participated. The HUNT3 cohort profile has been published previously [11].

Two sub-studies in HUNT3, the Lung Study and the Fitness Study, provided measurements from spirometry and CPET, respectively. Selection into the Lung Study included a 10% random sample in addition to subjects with self-reported asthma, chronic obstructive pulmonary disease (COPD), respiratory symptoms and use of medication for asthma/COPD. In total, 18,244 subjects were invited and 66.5% responded. Selection into the Fitness Study included subjects without self-reported respiratory symptoms, cardiorespiratory disease and medication for hypertension or asthma/COPD. From four selected municipalities, 12,609 subjects were eligible for CPET. At the end of the study 36.7% had completed the test [12].

The present study represents an overlap between the Lung Study and the Fitness Study including only subjects without self-reported respiratory symptoms or cardiopulmonary disease. Subjects aged 20 – 79 years (*n* = 918) were included. We excluded 28 subjects with FEV_1_ < LLN and 53 subjects with peak respiratory exchange ratio (RER_peak_) ≤ 1.00, indicating potential submaximal effort in healthy subjects. Furthermore, 96 subjects had missing values regarding covariates, heart rate reserve (HRR) or VE_peak_, leaving 741 subjects for the statistical analyses.

### Dynamic lung volume and ventilatory reserve

Spirometry (MasterScope Jaeger version 5.1; JAEGER, Wuerzburg, Germany) was conducted in accordance with the American Thoracic Society/European Respiratory Society recommendations [13]. FEV_1_ was chosen as a variable of dynamic lung volume because of high reproducibility and common use in clinical practice, and the LLN was defined as the fifth percentile (z-score = −1.645). Predicted values and Z-scores were calculated using the Global Lung Function Initiative 2012 (GLI-2012) software [14].

Ventilatory reserve was calculated as VR = 1 – VE_peak_/maximal voluntary ventilation (MVV). MVV was estimated by FEV_1_ × 40 [15]. VR was dichotomized into preserved (≥ 20%) and reduced (< 20%) levels [16].

### Oxygen uptake, oxygen pulse and heart rate reserve

CPET was performed on a treadmill (DK7830; DK City, Taichung City, Taiwan) with a facemask (Hans Rudolph, Shawnee, KS, USA) and a heart rate (HR) monitor (Polar S610/RS400; Polar Electro Oy, Kempele, Finland). A portable system (MetaMax II; CORTEX Biophysik GmbH, Leipzig, Germany) measured VO_2_, carbon dioxide output (VCO_2_) and VE. Incremental exercise was performed by an individualized protocol of increasing speed and/or incline until exhaustion as previously published [17].

Maximal oxygen uptake (VO_2max_), defined as RER_peak_ > 1.05 and VO_2_ increase < 2 ml/kg/min despite increased workload, was attained by 87.4%. As these predefined criteria were not met in all subjects, the term VO_2peak_ was used to designate the highest VO_2_ achieved. In the 12.6% (93 subjects) not meeting the VO_2max_ criteria, RER_peak_ and HRR were used to assess subject effort. No subjects had RER_peak_ ≤ 1.00 (exclusion criterion) and all but 8.6% (8 subjects) had HRR ≤ 5%.

VO_2peak_ was calculated as the mean of the three highest 10-s values and indexed by bodyweight (ml/kg/min). Peak oxygen pulse was calculated as peak O_2pulse_ (ml/beat) = VO_2peak_ (ml/min)/peak HR (beat/min). Heart rate reserve was calculated as HRR = 1 – peak HR/(220 – age).

### Covariates

Covariates were chosen based on a priori knowledge of associations with both the explanatory variable and the outcome variable. Age (years) was recorded at time of CPET. Body mass index (BMI) was calculated as the ratio of bodyweight (kg) and height squared (m^2^). Physical activity index (PAI) was calculated as the product of scores given to answers on questions of frequency (< once a week = 0, once a week = 1, two – three times a week = 2 and approximately every day = 3), duration (< 30 min = 1, ≥ 30 min = 1.5) and intensity of leisure time physical activity (Borg Rating of Perceived Exertion (RPE) 6 – 11 = 0, 12 – 13 = 5 and 14 – 20 = 10). Apart from redefining low, moderate and vigorous intensity as Borg RPE 6 – 11, 12 – 13 and 14 – 20, respectively [17], scores were assigned concordant with a non-exercise VO_2peak_ prediction model from HUNT3 [18]. Smoking status (never, former, daily and occasional) was obtained through questionnaire.

### Statistical analyses

Descriptive statistics of the total sample and the sample stratified by sex and VR were calculated as mean and standard deviation (SD) or number of observations and percentages unless otherwise specified. Continuous variables were compared using independent samples t tests.

The association between FEV_1Z-score_ and VO_2peak_ was modeled using linear regression. Regression coefficients with 95% confidence intervals (CI) were estimated in the total sample and in subgroups stratified by sex in three models. Model 1 estimated the crude association between FEV_1Z-score_ and VO_2peak_. Model 2 adjusted for BMI and smoking status. Model 3 additionally adjusted for age and PAI. Sex was included as a covariate along with an interaction term (FEV_1Z-score_ x sex) when men and women where combined in model 3. BMI and PAI were included as continuous variables and smoking status and age (categorized into 10-year age-groups) as categorical. To assess potential residual confounding by smoking, the association between FEV_1Z-score_ and VO_2peak_ was investigated in a subgroup including only never-smokers (*n* = 376) with adjustment for BMI, age, PAI and sex.

The association between FEV_1Z-score_ and VO_2peak_ was also assessed in subgroups stratified by dichotomized age (below and above 45 years) and adjusted for BMI, smoking status, PAI and sex.

The association between FEV_1Z-score_ and peak O_2pulse_ was assessed in the total sample and in subgroups stratified by sex. The estimates were adjusted for covariates equivalent to model 3 in the primary analyses. The interaction term (FEV_1Z-score_ x sex) was included when men and women were combined.

Model assumptions were assessed by residual plots and no violations were discovered. Statistical analyses were performed with IBM SPSS statistics 22.0 (IBM Corp., Armonk, NY, USA).

### Ethics

All subjects gave written informed consent. The study was approved by the Regional Committee for Medical and Health Research Ethics (REC Central 2015/1758).

## Results

Compared to women, men were of similar age, had similar BMI, reported similar level of physical activity, were less likely to report daily smoking, had similar HRR, lower VR, higher FEV_1Z-score_, higher VO_2peak_, higher peak O_2pulse_ and similar RER_peak_ (Table 1).

**Table 1:** Descriptive statistics stratified by sex

	Men (*n* = 359)	Women (*n* = 382)
Age median (years) (Interquartile range)	46.5 (36.5 – 55.5)	45.0 (32.6 – 55.2)
Age group (years)		
20 – 29	65 (18.1%)	77 (20.2%)
30 – 39	45 (12.5%)	71 (18.6%)
40 – 49	99 (27.6%)	86 (22.5%)
50 – 59	91 (25.3%)	97 (25.4%)
60 – 69	48 (13.4%)	44 (11.5%)
70 – 79	11 (3.1%)	7 (1.8%)
BMI (kg/m^2^)	26.5 ± 3.0	25.4 ± 3.8
PAI median (Interquartile range)	15.0 (0.0 – 30.0)	15.0 (7.5 – 30.0)
Smoking status		
Never	186 (51.8%)	190 (49.7%)
Former	109 (30.4%)	122 (31.9%)
Daily	24 (6.7%)	40 (10.5%)
Occasional	40 (11.1%)	30 (7.9%)
HRR	−0.05 ± 0.08	−0.04 ± 0.07
VR	0.23 ± 0.12	0.30 ± 0.11
FEV_1Z-score_	0.28 ± 0.83	0.19 ± 0.87
VO_2peak_ (ml/kg/min)	45.8 ± 9.1	36.8 ± 7.6
Peak O_2pulse_ (ml/beat)	21.3 ± 3.8	14.0 ± 2.4
RER_peak_	1.14 ± 0.05	1.13 ± 0.06

*BMI* body mass index, *PAI* physical activity index, *HRR* heart rate reserve, *VR* ventilatory reserve, *FEV*_*1Z-score*_ forced expiratory volume in one second Z-score, *VO*_*2peak*_ peak oxygen uptake, *O*_*2pulse*_ oxygen pulse, *RER*_*peak*_ peak respiratory exchange ratio

The proportions of subjects with reduced VR were 33.4% (men) and 13.6% (women). Age and BMI were similar in all subgroups stratified by sex and VR. In both sexes, subjects with preserved VR were less active than subjects with reduced VR. The HRR was negative and RER_peak_ similar in all subgroups (Table 2).

**Table 2:** Descriptive statistics stratified by sex and ventilatory reserve (VR)

	Men		Women	
	VR ≥ 20% (*n* = 239)	VR < 20% (*n* = 120)	VR ≥ 20% (*n* = 330)	VR < 20% (*n* = 52)
Age median (years) (interquartile range)	46.0 (35.7 – 55.7)	46.7 (38.1 – 54.8)	44.1 (31.6 – 54.6)	47.4 (35.0 – 57.8)
BMI (kg/m^2^)	26.4 ± 3.1	26.8 ± 2.8	25.2 ± 3.7	26.8 ± 3.9
PAI median (interquartile range)	15.0 (0.0 – 30.0)	30.0 (8.1 – 30.0)	15.0 (7.5 – 30.0)	22.5 (10.0 – 30.0)
HRR	−0.05 ± 0.08	−0.05 ± 0.08	−0.04 ± 0.07	−0.04 ± 0.07
FEV_1Z-score_	0.47 ± 0.82	−0.09 ± 0.72	0.27 ± 0.85	−0.32 ± 0.77
VO_2peak_ (ml/kg/min)	44.1 ± 8.5	49.1 ± 9.3	36.7 ± 7.5	37.9 ± 8.2
Peak O_2pulse_ (ml/beat)	20.5 ± 3.5	22.9 ± 3.9	13.7 ± 2.3	15.4 ± 3.0
RER_peak_	1.14 ± 0.05	1.15 ± 0.05	1.13 ± 0.06	1.14 ± 0.06

*BMI* body mass index, *PAI* physical activity index, *HRR* heart rate reserve, *FEV*_*1Z-score*_ forced expiratory volume in one second Z-score, *VO*_*2peak*_ peak oxygen uptake, *O*_*2puls*e_ oxygen pulse, *RER*_*peak*_ peak respiratory exchange ratio

Compared to subjects with preserved VR, subjects with reduced VR had lower FEV_1Z-score_ in both men (difference 0.57, 95% CI 0.39 – 0.74) and women (difference 0.59, 95% CI 0.35 – 0.84), higher VO_2peak_ in both men (difference 5.0 ml/kg/min, 95% CI 3.1 – 6.9) and women (difference 1.2 ml/kg/min, 95% CI -1.0 – 3.5) and higher peak O_2pulse_ in both men (difference 2.4 ml/beat, 95% CI 1.6 – 3.2) and women (difference 1.6 ml/beat, 95% CI 0.7 – 2.5).

In all three models, FEV_1Z-score_ was positively associated with VO_2peak_ (Table 3). In women the point estimates (β) were similar in model 2 (β = 1.6) and model 3 (β = 1.6). In men the point estimate in model 2 (β = 0.3) was lower than in model 3 (β = 0.9). In model 3, the association between FEV_1Z-score_ and VO_2peak_ did not differ statistically by sex (*p*-value for interaction = 0.16).

**Table 3:** The association between FEV_1Z-score_ and VO_2peak_ (ml/kg/min) in total sample and stratified by sex

		Model 1^a^		Model 2^b^		Model 3^c^	
	n	β	95% CI	β	95% CI	β	95% CI
Total	741	1.8	1.0 – 2.6	1.3	0.6 – 2.0	1.2	0.7 – 1.6
Men	359	0.8	−0.4 – 1.9	0.3	−0.7 – 1.3	0.9	0.2 – 1.6
Women	382	2.2	1.3 – 3.0	1.6	0.9 – 2.3	1.6	1.0 – 2.1

*FEV*_*1Z-score*_ forced expiratory lung volume in one second Z-score, *VO*_*2peak*_ peak oxygen uptake, *β* regression coefficient for FEV_1Z-score_

^a^Crude model. ^b^Adjusted for body mass index (BMI) and smoking status. ^c^Adjusted for BMI, smoking status, age, physical activity index (PAI) with addition of sex in total sample model

In the sensitivity analysis including only never-smokers, FEV_1Z-score_ was positively associated with VO_2peak_ (β = 0.9, 95% CI 0.2 – 1.5), as in the main model.

Stratified by dichotomized age, FEV_1Z-score_ was positively associated with VO_2peak_ in both categories (Table 4).

**Table 4:** The association between FEV_1Z-score_ and VO_2peak_ (ml/kg/min) in total sample and stratified by dichotomized age

	n	β	95% CI
Total	741	1.2	0.7 – 1.6
Age < 45 years	357	1.2	0.5 – 1.8
Age ≥ 45 years	384	1.2	0.5 – 1.9

*FEV*_*1Z-score*_ forced expiratory lung volume in one second Z-score, *VO*_*2peak*_ peak oxygen uptake, *β* regression coefficient for FEV_1Z-score_ adjusted for body mass index (BMI), smoking status, physical activity index (PAI) and sex with addition of age in total sample model

FEV_1Z-score_ was positively associated with peak O_2pulse_ in both women and men (Table 5). The association did not differ statistically by sex (p-value for interaction = 0.67).

**Table 5:** The association between FEV_1Z-score_ and peak O_2pulse_ (ml/beat) in total sample and stratified by sex

	n	β	95% CI
Total	741	0.33	0.10 – 0.56
Men	359	0.32	−0.08 – 0.73
Women	382	0.39	0.14 – 0.63

*FEV*_*1Z-score*_ forced expiratory lung volume in one second Z-score, *O*_*2pulse*_ oxygen pulse, *β* regression coefficient for FEV_1Z-score_ adjusted for body for mass index (BMI), smoking status, age, physical activity index (PAI) with addition of sex in total sample model

## Discussion

The main finding in this study was a positive association between FEV_1_ above LLN and VO_2peak_ in healthy subjects, aged 20 – 79 years, where the majority had preserved VR. The association was preserved after performing sub-analyses in both men and women, in never-smokers and in subjects below and above 45 years of age.

This is one of the first studies to investigate the association between normal dynamic lung volume and VO_2peak_. Hassel et al. [8] reported positive associations between resting measures of lung function within the normal range and VO_2peak_ in healthy subjects aged 69 – 77 years. The present study expands on this work by assessing a more general population including young and middle-aged subjects. The results show that age-induced reduction in dynamic lung volume is not likely to fully explain the positive association between FEV_1Z-score_ and VO_2peak_.

Although the results show an association between FEV_1Z-score_ and VO_2peak_, potential mechanisms are unclear. The majority of men (66.6%) and women (86.4%) had preserved VR, indicating residual capacity to increase ventilation at peak exercise. Preserved VR is commonly observed in healthy non-athletes performing maximal exercise at sea level, reflecting large ventilatory capacity relative to metabolic demand. Furthermore, the normal physiological response at peak exercise is characterized by low HRR, indicating maximal utilization of cardiovascular capacity for oxygen transport. Combined, preserved VR and low HRR characterize cardiovascular limitation [5]. As this pattern was observed in the majority of subjects, the positive association between FEV_1Z-score_ and VO_2peak_ does not seem to be conditional on ventilatory limitation. Additionally, we found a positive association between FEV_1Z-score_ and peak O_2pulse_ in both sexes. As O_2pulse_ is a proposed non-invasive estimator of cardiac stroke volume, we cannot rule out an influence of normal dynamic lung volume on cardiac output in healthy non-athletes. Although such a physiological interaction between the pulmonary and the cardiac system remains hypothetical, it may be appropriate to consider measures of dynamic lung volumes as covariates in future studies of exercise performance in healthy subjects.

Reduced VR is due to reduced ventilatory capacity and/or increased ventilatory demand, reflecting two different mechanisms for ventilatory limitation to exercise. Reduced VR secondary to reduced ventilatory capacity is commonly observed in COPD, where reduced dynamic lung volume with expiratory flow limitation and dynamic hyperinflation are important mechanisms of exercise intolerance [19]. Although FEV_1Z-score_ was lower in subjects with reduced VR, all subjects had FEV_1_ above LLN. Furthermore, exercise intolerance due to ventilatory limitation in COPD is reflected by low VO_2peak_ [5] and high HRR indicating residual capacity of the cardiac system at peak exercise. In contrast, we found higher VO_2peak_ in men and women with reduced VR, suggesting higher CRF in these subjects. Additionally, all subgroups had negative HRR, reflecting a physiological response with cardiovascular limitation independent on VR. Accordingly we do not consider mechanical ventilatory limitation likely to explain the association between FEV_1Z-score_ and VO_2peak_ in healthy subjects with FEV_1_ above LLN.

In contrast to reduced VR from reduced ventilatory capacity, reduced VR from increased ventilatory demand is observed in healthy endurance trained subjects with high CRF [5]. High CRF with high VO_2peak_ may be associated with respiratory muscle fatigue [20], and affect gas exchange if the upper limits for oxygen transport by the pulmonary system are exceeded [21]. Exercise induced arterial hypoxemia (EIAH) reduces VO_2peak_ in significant proportions of endurance trained athletes [22], but is not usually observed in healthy subjects from a general population with lower VO_2peak_. Therefore, EIAH is unlikely to explain the positive association between FEV_1Z-score_ and VO_2peak_ found in this study.

Although mechanisms associated with mechanical ventilatory limitation and EIAH are unlikely to explain the association between FEV_1Z-score_ and VO_2peak_, we cannot exclude this possibility. Stratification on dichotomized VR was considered but not performed, as VR is a likely collider on the association between FEV_1Z-score_ and VO_2peak_ potentially inducing biased estimates in stratified analyses. Furthermore, FEV_1_ may be a marker of non-uniform ventilation due to small airway and/or parenchymal disease/properties causing increased heterogeneity in ventilation-perfusion relationships and variability in gas exchange, which is not excluded by preserved VR. Accordingly, our study raises further questions that potentially impact current understanding of respiratory and exercise physiology. Future studies should focus on uncovering the underlying mechanisms of the associations we report, and are likely to benefit from including invasive measures from arterial blood gases.

The strengths of this study include the high number of subjects from a general population. Direct measurement of VO_2_ is the gold-standard for evaluating CRF and was used in this study. Furthermore, we used the GLI-2012 reference equations, found valid for the Norwegian population [23], taking into account age, sex and height related variance in FEV_1_. Although we did not a priori consider age and PAI as confounders of the association between FEV_1Z-score_ and VO_2peak_, these variables are known predictors of VO_2peak_ [18], and were included as covariates in the final models. Confounding was detected in men and accordingly adjusted for. Although we can only test for associations, we consider an influence of both FEV_1Z-score_ on VO_2peak_ and FEV_1Z-score_ on peak O_2pulse_ more likely than vice versa, thereby reducing the limitations from the cross-sectional study design.

There are some limitations. The external validity may suffer, as subjects recruited to a fitness study may be more active and fit than the general population. All subjects were considered to be healthy, but eligibility for the HUNT Fitness study was evaluated only through self-report. Although secondary criteria of maximal subject effort were met, measured VO_2peak_ may have underestimated true VO_2max_ in the 12.6% not meeting the VO_2max_ criteria [24], potentially influencing the precision of the estimates. Additionally, lack of measurements from arterial blood gases limits further discussion on potential physiological mechanisms related to variability in gas exchange. Finally, O_2pulse_ is an indirect approximate of cardiac stroke volume, and the association between FEV_1Z-score_ and peak O_2pulse_ permits only speculation on a potential influence of normal dynamic lung volume on cardiac performance during exercise.

## Conclusions

Normal dynamic lung volume, defined as FEV_1_ above LLN, was positively associated with VO_2peak_ in both men and women, in never-smokers and in subjects below and above 45 years of age. The majority of subjects had preserved VR, and the results suggest that FEV_1_ within normal limits may influence VO_2peak_ in healthy subjects even when no ventilatory limitation to exercise is evident. Future studies should focus on understanding the underlying mechanisms of this association. If causality can be established, it would further emphasize the importance of respiratory health.

## Abbreviations

BMI: Body mass index
CI: Confidence interval
COPD: Chronic obstructive pulmonary disease
CPET: Cardiopulmonary exercise test
CRF: Cardiorespiratory fitness
EIAH: Exercise induced arterial hypoxemia
FEV_1_: Forced expiratory lung volume in one second
GLI-2012: Global Lung Function Initiative 2012
HR: Heart rate
HRR: Heart rate reserve
HUNT: The Nord-Trøndelag Health Study
LLN: Lower limit of normal
MVV: Maximal voluntary ventilation
O_2pulse_: Oxygen pulse
PAI: Physical activity index
REC: Regional Committee for Medical and Health Research Ethics
RER_peak_: Peak respiratory exchange ratio
RPE: Rating of percieved exertion
SD: Standard deviation
VCO2: Carbon dioxide output
VE_peak_: Peak minute ventilation
VO_2max_: Maximal oxygen uptake
VO_2peak_: Peak oxygen uptake
VR: Ventilatory reserve
